# The complete chloroplast genome of *Dendrocalamus hamiltonii* (Poaceae, Bambuseae)

**DOI:** 10.1080/23802359.2020.1772696

**Published:** 2020-06-08

**Authors:** Ruli Zhang, Yu-Xiao Zhang, Weiyi Liu, Chaomao Hui

**Affiliations:** a Sympodial Bamboos Technological and Engineering Research Center (SymBTERC) National Forestry and Grassland Administration (NFGA), Southwest Forestry University, Kunming, China; b Institute of Bamboo and Rattan, Southwest Forestry University, Kunming, China; c Yunnan Academy of Biodiversity, Southwest Forestry University, Kunming, China

**Keywords:** *Dendrocalamus hamiltonii*, chloroplast genome, phylogenomic

## Abstract

Dendrocalamus *hamiltonii* is one of the best bamboo species with bamboo shoots, and has higher economic value. The chloroplast genome is a circular molecule of 139404 bp in length, consisting of a 82938 bp large single copy region (LSC), a 12876 bp small single copy region (SSC), and a pair of inverted repeats region (IRa and IRb: 21795 bp each). The GC content of chloroplast genome is 38.9%. The cp genome contains a total of 133 genes, including 86 protein-coding genes, 8 rRNA genes, and 39 tRNA genes. Moreover, phylogenomic analysis showed that *D. hamiltonii* and *D. brandisii* clustered together in one branch.

*Dendrocalamus hamiltonii* is a large bamboo species with sympodial rhizome that is classified as genus *Dendrocalamus* within the family Gramineae and subfamily Bambusoideae, and is widely cultivated in Xishuangbanna, Puer and Lincang, Yunnan province, in China. It is known as the three famous sweet bamboo shoots in the world with *D. brandisii* and *D. asper.* The young shoots are sweet and delicious, which are used as vegetables. It is also high-quality building material used by national minority to build houses (Wang et al. 2019; Yang et al. 2019). In this study, we used Illumina Hiseq sequencing, the chloroplast complete genome of *D. hamiltonii* was obtained, to provide evidence that the research on the phylogeny of *Dendrocalamus.*

The fresh leaves were collected in 2019 from Southwest Forestry University (25° 6′30″N, 102° 45′23″E), China. The specimen was deposited at Institute of Bamboo and Rattan, Southwest Forestry University (specimen number:SWFU1992015). Total DNA of *D. hamiltonii* was extracted by Rapid Plant Genomic DNA Isolation Kit (BALB, Beijing, China). Paired-end reads were sequenced by using Illumina Hiseq platform in Sangon Biotech in Shanghai, China, which gained 4.4 Gb of 150 bp paired-end raw reads. The cp genome of *D. hamiltonii* was assembled using the program GetOrganelle (Jin et al. 2018), and the assembled cp genome was annotated using CPGAVAS2(Shi et al. 2019) based on *D. brandisii* cp genome (GenBank Accession: MN782325). To determine the phylogenetic relationship of *D. hamiltonii*, based on 29 cp genomes within the subfamily Bambusoideae, and were downloaded from NCBI. All cp genomes were aligned using the program MAFFT v7.450 (Rozewicki et al. 2019), and phylogenetic tree (maximum likelihood) constructed by RAxML-NG v0.90 (Kozlov et al. 2019) with 1000 bootstrap replicates, best-fitted model was confirmed is TIM1 + I + G4 by ModelTest-NG (Darriba et al. 2019).

The cp genome of *D. brandisii* (GenBank Accession:MN933913) is a circular molecule of 139404 bp in length with GC average content is 38.9%, and consisting of a 82938 bp large single copy region (LSC), a 12876 bp small single copy region (SSC), and two inverted repeats region of 21795 bp. The cp genome of *D. hamiltonii* contains a total of 133 genes, including 86 protein-coding genes, 8 rRNA genes, and 39 tRNA genes, and the introns were found in 13 genes, six protein-coding genes (rps16, atpF, rpl16, rpl2, ndhB, ndhA) and six tRNA genes (trnK-UUU, trnG-UCC, trnL-UAA, trnV-UAC, trnI-GAU, trnA-UGC) have one intron each, one gene (ycf3) has two introns. The phylogenomic analysis reveals that *D. hamiltonii* clustered together with *D. brandisii* (Figure 1). Five samples of *Dendrocalamus* that constituted a monophyletic, and was sister to *Bambusa* within Bambusoideae.

**Figure 1.**
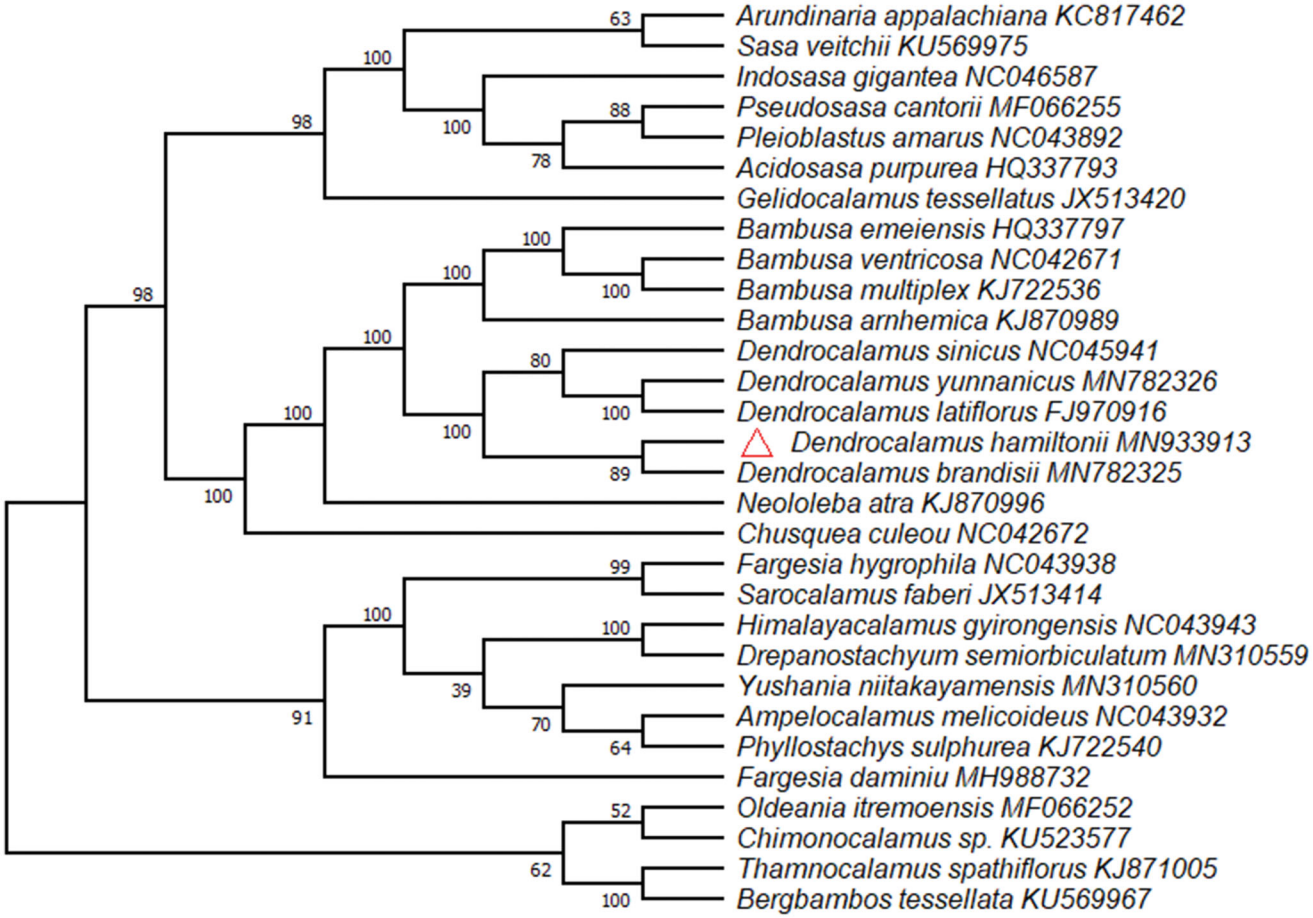
The maximum likelihood phylogenetic tree constructed from 30 species chloroplast genomes. The numbers near the branch are the bootstrap support value.

## Data Availability

My data has been uploaded to NCBI (https://www.ncbi.nlm.nih.gov/). GenBank Accession:MN933913.
